# ﻿Three new species and five new records within the genus *Lilioceris* (Coleoptera, Chrysomelidae, Criocerinae) from China

**DOI:** 10.3897/zookeys.1189.111064

**Published:** 2024-01-12

**Authors:** Yuan Xu, Hongbin Liang

**Affiliations:** 1 Key Laboratory of Zoological Systematics and Evolution, Institute of Zoology, Chinese Academy of Sciences, Beijing 100101, China Institute of Zoology, Chinese Academy of Sciences Beijing China; 2 College of Life Science, University of Chinese Academy of Sciences, Beijing 100049, China University of Chinese Academy of Sciences Beijing China

**Keywords:** Habitat, host plant, map, new synonym, Shining leaf beetle, taxonomy, Tibet

## Abstract

The Chinese species of *Lilioceris* are revised, and three new species are described from Tibet, China: *Lilioceriszhentangensis* Xu & Liang, **sp. nov.**, *Liliocerismedogensis* Xu & Liang, **sp. nov.** and *Lilioceriszayuensis* Xu & Liang, **sp. nov.** Five species of *Lilioceris* are reported for China as new records: *L.dromedarius* (Baly, 1861), *L.pulchella* (Baly, 1859), *L.semicostata* (Jacoby, 1908), *L.unicolor* (Hope, 1831) and *L.nepalensis* Takizawa, 1989. *Liliocerisseminigra* (Jacoby, 1889) is proposed as a junior synonym of *L.unicolor* Hope, 1831. Redescriptions, habitus photographs, geographic distributions, host plants (if available) and habitats are provided for these species.

## ﻿Introduction

*Lilioceris* Reitter, 1913 is the second largest genus of Criocerinae, containing approximately 160 species in the world and 60 species in China. Most of the species of *Lilioceris* are distributed in the Oriental region (Clavareau 1913; Monrós 1960) and the Chinese species are mainly distributed in the southern part of China (Bezděk and Schmitt 2017).

Tibet is the second largest autonomous region in China, with various climatic zones from the tropics to frigid mountains. However, *Lilioceris* in Tibet has been poorly investigated in the past: only four species have been recorded (Yang 2004). *Liliocerissubpolita* (Motschulsky) was obviously misidentified from Tibet and is excluded from its fauna (Bezděk and Schmitt 2017; Xu et al. 2021). In recent years seven expeditions were made in Tibet and Yunnan. These expeditions resulted in more than two thousand specimens of Criocerinae. Among *Lilioceris* collected in Dinggyê, Mêdog and Zayü were species new to science. We also identified five species, collected in Tibet and Yunnan that were originally recorded in India, Nepal or Cambodia.

The purpose of this paper is three-fold: to describe three new species from Tibet, to report five new distribution records from Tibet, Yunnan and Hainan, and to synonymize a species of *Lilioceris*.

## ﻿Material and methods

Specimens from several museums and collections were examined. The collections cited in this article are indicated by the following abbreviations: **IZCAS** = National Zoological Museum, Institute of Zoology, Chinese Academy of Sciences, Beijing, China; **MNHN** = Museum national d’Histoire naturelle, Paris, France; **NHML** = Natural History Museum, London, UK; **SEHU** = Systematic Entomology, Graduate School of Agriculture, Hokkaido University, Japan.

Dry specimens were soaked in hot water for 1–2 hours. Then the abdomen was opened at its latero-apical margin and genitalia removed using forceps, soaked in warm 10% KOH for 1 h, and dyed in Chlorazol Black E. The basal orifice of the aedeagus was injected with 100% ethanol with a micro-injector until the internal sac was fully everted. The aedeagus with its everted internal sac was photographed using a large depth-of-field 3D digital microscope (Keyence VHX–1000C) and edited in Adobe Photoshop (CC). For storage, a microvial with genitalia was pinned to the specimen from which the genitalia were removed.

Body length (**BL**) was measured from the anterior margin of the labrum to the apex of the elytra; body width (**BW**) was measured along the greatest elytral width.

Other methods of specimen observation and preparation follow previous publications (Tishechkin et al. 2011; Li et al. 2013). Morphological terminology follows Chou et al. (1993) and Matsumura et al. (2013). Redescriptions are provided for newly recorded species because of their insufficient original information.

## ﻿Taxonomic account

### ﻿ Lilioceris zhentangensis

Taxon classificationAnimaliaColeopteraChrysomelidae

Xu & Liang sp. nov.

4E60EB66-A03C-5247-B8BE-E1378C7E606A

https://zoobank.org/1AAF4F83-3597-4A54-BE59-5BA316F1D980

Figs 1 , 2 , 19A–D , 22A–D , 28A–C , 36 , 42–45

#### Material examined.

Total 47 specimens. ***Holotype***: 1♂, Tibet, Dinggyê, Zhêntang, Nadang village / 2021.6.25 / 27.85317°N, 87.44903°E, 2491 m / Hongbin Liang, Yuan Xu and Neng Zhang coll. (IZCAS); ***Paratypes***: 7♀9♂, Tibet, Dinggyê, Zhêntang, Nadang village / 2021.6.25 / 27.85317°N, 87.44903°E, 2491 m / Hongbin Liang, Yuan Xu and Neng Zhang coll. (IZCAS); 3♀1♂, Tibet, Dinggyê, Zhêntang, Jiuyan Hot Spring / 2021.6.24 / 27.9068°N, 87.3777°E, 2704 m / Hongbin Liang, Yuan Xu and Neng Zhang coll. (IZCAS); 1♀2♂, Tibet, Dinggyê, Zhêntang, Qizi Tang / 2021.6.23 / 27.91232°N, 87.38273°E, 2619 m / Hongbin Liang, Yuan Xu and Neng Zhang coll. (IZCAS); 3♀6♂, Tibet, Nyingchi, Lunang road, Dongjiu village / 2022.7.23 / 29.913910°N, 94.798072°E, 2643 m / Hongbin Liang, Yuan Xu and Neng Zhang coll. (IZCAS); 3♀5♂, Tibet, Nyingchi, Lunang, Baimu village / 2022.7.24 / 29.988540°N, 94.746077°E, 2622 m / Hongbin Liang, Yuan Xu and Neng Zhang coll. (IZCAS); 2♀2♂, Tibet, Nyingchi, Lunang, Baga village / 2022.7.24 / 29.998361°N, 94.695714°E, 2771 m / Hongbin Liang, Yuan Xu and Neng Zhang coll. (IZCAS); 2♀, Tibet, Bomi, Yi’ong, Bayu village / 2022.7.25 / 30.334625°N, 94.804114°E, 2296 m / Hongbin Liang, Yuan Xu and Neng Zhang coll. (IZCAS).

#### Diagnosis.

Antennae nearly half as long as body length, antennomeres V–X quadrate. Pronotum with distinct anterior and posterior transverse impressions, pronotal disc with two rows of fine and irregular punctures in middle. Elytral punctures sparse on basal 2/3 and absent on apical 1/3. Lateroposterior corner of metasternum densely pubescent.

#### Description.

BL = 7.0–9.0 mm, BW = 3.3–4.2 mm. Head, legs, scutellum, mesosternum, lateral metasternum, and metepisternum black; pronotum, elytra, middle metasternum, and abdomen brownish red.

**Figures 1–4.**
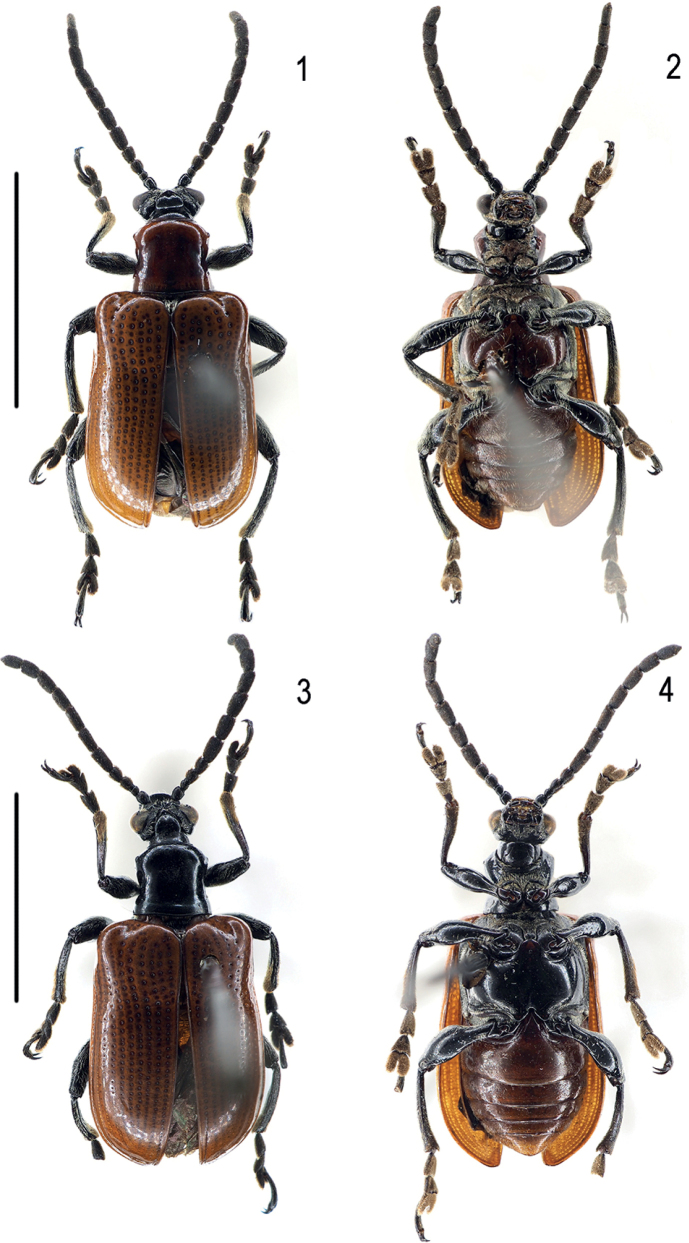
Habitus of new *Lilioceris* species (holotypes) **1, 2***L.zhentangensis*, holotype, China (Tibet) **3, 4***L.medogensis*, holotype, China (Tibet). Scale bars: 5.0 mm.

***Head*** (Fig. 1). Vertex with shallow groove in middle, punctate and pubescent, almost smooth; frontoclypeal area triangular, lateral side of disc with sparse punctures and pubescence; labrum transverse, with sparse long pubescence; antennae nearly half as long as body, antennomeres I–IV nearly globular, antennomeres V–X 1.2 times as long as wide, III–XI densely pubescent and punctate.

***Pronotum*** (Figs 1, 19A). Anterior angle protruding, posterior angle not protruding; sides distinctly constricted in middle; anterior and posterior transverse impression distinct; middle of disc with two rows of fine and irregular punctures; basal transverse groove indistinct. Scutellum triangular and densely pubescent.

***Elytra*** (Fig. 1). Humeri protruding, humeral groove shallow, basal impression distinct; striae with large punctures at base, punctures diminished posteriorly and absent on apical 1/3, intervals without punctures; epipleura raised, with row of fine punctures.

***Mesosternum*** pubescent; apical portion of mesosternal process narrow and flat, obliquely pointed, not horizontally connected with metasternum. Metasternal disc very sparsely pubescent, lateroposterior corner with short strip of pubescence. Metepisternum densely pubescent (Fig. 19B).

***Abdominal sternites*** (Fig. 19C). Lateral transverse impressions distinct on sternites I–IV, area of transverse impressions and middle of sternites I–IV smooth, other areas with dense pubescence and punctures.

***Legs*** (Fig. 2). Femora with dense pubescence on dorsal surface, with sparse pubescence on ventral surface, middle area widened.

***Male genitalia*** (Fig. 22A–D). Apical foramen occupying 1/5 length of median lobe (Fig. 22A); apex hooked (Fig. 22B); tegmen Y-shaped, basal piece of tegmen oval and broad, lateral lobes slightly sclerotized and combined with second connecting membrane; internal sac with distinct dorsal and ventral sclerites, posterior part of dorsal sclerite in dorsal view parallel, ventral sclerite extended and tubular, median sclerite very small (Fig. 22C, D).

***Female reproductive organs*** (Fig. 28A–C). Tergites VIII and IX, and sternites VIII and IX of female sclerotized, posterior areas of tergite VIII and sternite VIII with pubescence and apodemes, spiculum gastrale Y-shaped and long; ovipositor with dense pubescence, distal part of ovipositor cylindrical, short, with protuberance; spermatheca simple and curved.

#### Distribution

**(Fig. 36).** China (Tibet).

#### Etymology.

The specific name *zhentangensis* refers to its type locality Zhêntang, Dinggyê County, Tibet.

#### Host plant and habitat

**(Figs 42–45).** The host plant is *Smilaxmenispermoidea* A. DC., (Smilacaceae) according to our observations in Zhêntang town. Zhêntang is located in a deep valley at the southern part of the Himalayas. Warm and humid air currents from the Indian Ocean enter the valley frequently. Abundant rainfall and rugged topography in the valley make the biodiversity of Zhêntang very rich. This species occurs at the altitude of 2200 to 2800 m. The habitat is open, composed of tall trees, woody vines and weeds.

#### Remarks.

This species looks similar to *L.cyanicollis* (Pic, 1916) (our concept is based on a specimen determined by J. L. Gressitt, NHML), but is differentiated by its pronotum with distinct anterior and posterior transverse impressions; head, antennae, and legs without blue metallic luster. In *L.cyanicollis*, the pronotum only with a weak posterior transverse impression; head, antennae, and legs with a blue metallic luster. It is also similar to *L.latissima* (Pic, 1932) (based on a syntype studied, MNHN), but differs by the metasternum with a short strip of pubescence. In *L.latissima*, the metasternum is glabrous.

### ﻿ Lilioceris medogensis

Taxon classificationAnimaliaColeopteraChrysomelidae

Xu & Liang sp. nov.

836380FB-7554-5DB9-8BBF-74D0D2F50E1D

https://zoobank.org/88E1CB44-7DD5-47FF-9373-D6A0679F670A

Figs 3 , 4 , 20A–D , 23A–D , 29A–C , 36 , 38–41

#### Material examined.

Total 51 specimens. ***Holotype***: 1♂, Tibet, Mêdog, Renqingbung temple / 2020.9.3 / 29.30564°N, 95.35326°E, 1982 m / Hongbin Liang and Neng Zhang coll. (IZCAS); ***Paratypes***: 7♀7♂, Tibet, Mêdog, Renqingbung temple / 2020.9.3 / 29.30564°N, 95.35326°E, 1982 m / Hongbin Liang and Neng Zhang coll. (IZCAS); 4♀5♂, Tibet, Mêdog, Renqingbung temple / 2020.9.12 / 29.30564°N, 95.35326°E, 1982 m / Hongbin Liang and Neng Zhang coll. (IZCAS); 2♀4♂, Tibet, Mêdog, Renqingbung temple / 2021.6.9 / 29.30564°N, 95.35326°E, 1982 m / Hongbin Liang, Yuan Xu and Neng Zhang coll. (IZCAS); 3♀8♂, Tibet, Mêdog, Renqingbung temple / 2022.7.18 / 29.30564°N, 95.35326°E, 1982 m / Hongbin Liang, Yuan Xu and Neng Zhang coll. (IZCAS); 5♀5♂, Tibet, Mêdog, Renqingbung temple / 2023.7.13 / 29.30564°N, 95.35326°E, 1982 m / Neng Zhang coll. (IZCAS); 1♂, Tibet, Mêdog, Baibung, Dergong village / 2019.8.12, 29.19711°N, 95.14767°E, 1529 m / Hongbin Liang and Yuan Xu coll. (IZCAS); 1♂, Tibet, Mêdog, Baibung, Dergong village / 2022.7.17, 29.180592°N, 95.143494°E, 1656 m / Yuan Xu coll. (IZCAS); 1♂, Tibet, Mêdog, Baibung, Gelin village / 2019.8.12 29.22012°N, 95.17479°E, 1652 m / Hongbin Liang and Yuan Xu coll. (IZCAS); 4♀4♂, Tibet, Mêdog, Baibung, 11 km on Gelin road / 2021.6.11–15, 29.23370°N, 95.17707°E, 1408 m / Hongbin Liang, Yuan Xu and Neng Zhang coll. (IZCAS); 1♂, Tibet, Mêdog, Baibung Tea farm / 2020.9.7, 29.26310°N, 95.20983°E, 1047 m / Hongbin Liang coll. (IZCAS); 1♂, Tibet, Mêdog, Baibung, Ani bridge / 2023.7.15 / 29.315211°N, 95.175172°E, 923 m / Neng Zhang coll. (IZCAS).

#### Diagnosis.

Elytra and abdomen brownish red, rest of body black. Antennae nearly half as long as body length, antennomeres V–X quadrate. Pronotal disc with two rows of fine punctures in middle. Elytral punctures sparse and absent on apical 1/3.

#### Description.

BL = 8.0–10.0 mm, BW = 3.5–4.5 mm. Body black except elytra and abdomen brownish red.

***Head*** (Fig. 3). Vertex with shallow groove in middle, punctate and pubescent sparsely; frontoclypeal area triangular, lateral side of disc with sparse punctures and pubescence; labrum transverse, with sparse long pubescence; antennae nearly half as long as body, antennomeres I–IV nearly globular, antennomere II shortest, antennomeres V–X 1.2 times as long as wide, V–XI densely pubescent and punctate.

***Pronotum*** (Figs 3, 20B). Anterior angle protruding, posterior angle not protruding; sides distinctly constricted in middle; middle of disc with two rows of fine punctures; anterior and posterior transverse impression shallow, basal transverse groove indistinct. Scutellum triangular and densely pubescent.

***Elytra*** (Fig. 3). Humeri protruding, humeral groove and basal impression distinct; striae with large punctures at base, punctures diminished posteriorly and absent on apical 1/3, intervals without punctures; epipleura raised, with row of fine punctures.

***Mesosternum*** pubescent; apical portion of mesosternal process narrow and flat, obliquely pointed, not horizontally connected with metasternum. Metasternal disc almost glabrous, posterior margin with sparse pubescence (Fig. 20B). Metepisternum densely pubescent.

***Abdominal sternites*** (Fig. 20C). Lateral transverse impressions distinct on sternites I–IV. Lateral side of sternite I–IV and pygidium densely pubescent, other areas with sparse pubescence and punctures.

***Legs*** (Fig. 4). Femora with dense pubescence on dorsal surface, sparse pubescence on ventral surface, middle area widened.

***Male genitalia*** (Fig. 23A–D). Apical foramen occupying 1/5 length of median lobe (Fig. 23A); apex hooked (Fig. 23B); tegmen Y-shaped, basal piece of tegmen oval and broad, lateral lobes slightly sclerotized and combined with second connecting membrane; internal sac with distinct dorsal and ventral sclerites, posterior part of dorsal sclerite in dorsal view widened, ventral sclerite extended and tubular, median sclerite very small (Fig. 23C, D).

***Female reproductive organs*** (Fig. 29A–C). Tergites VIII and IX, and sternites VIII and IX sclerotized, posterior areas of tergite VIII and sternite VIII with pubescence and apodemes, spiculum gastrale Y-shaped and short; ovipositor with dense pubescence, distal part of ovipositor cylindrical, short and with protuberance; spermatheca simple and curved.

#### Distribution

**(Fig. 36).** China (Tibet).

#### Etymology.

The specific name *medogensis* refers to its type locality Mêdog, Tibet, China.

#### Host plant and habitat

**(Figs 38–41).** Beetles were found to feed on *Smilaxferox* Wall. Ex Kunth (Smilacaceae) in Mêdog in the northernmost edge of the tropics (China, Tibet, Mêdog), with altitudes of ~ 1000 to 2000 m. The type locality Renqingbung temple is located on a mountain in Mêdog County, with high temperatures, high humidity and plentiful precipitation. Vegetation type is subtropical evergreen broadleaved forest.

#### Remarks.

*Liliocerismedogensis* sp. nov. and *L.zhentangensis* sp. nov. are similar in their pronotia having anterior and posterior transverse depressions, which are easily distinguished from those of the other members of *Lilioceris*. However, *L.medogensis* sp. nov. is different from *L.zhentangensis* sp. nov. by the brownish red pronotum (Fig. 20A) and metasternum (Fig. 20B), only the lateral side of the metasternum is black; the lateroposterior corner of the metasternum is glabrous; and the posterior part of the dorsal sclerite of the male genitalia in dorsal view is widened (Fig. 23C). In *L.zhentangensis*, the pronotum and metasternum are black (Fig. 19A); the lateroposterior corner of the metasternum has a strip of pubescence (Fig. 19B); and the posterior part of the dorsal sclerite in dorsal view is parallel (Fig. 22C). In addition, their host plants and habitats are also different: *L.medogensis* lives on *Smilaxferox* in warmer and lower altitudes, while *L.zhentangensis* inhabits *Smilaxmenispermoidea* in colder, higher altitudes.

### ﻿ Lilioceris zayuensis

Taxon classificationAnimaliaColeopteraChrysomelidae

Xu & Liang sp. nov.

6151B602-3F24-5375-87C6-182A3E8EDCCD

https://zoobank.org/8D02C295-41CB-4A11-8A6A-88C5F4861D67

Figs 5 , 6 , 21A–D , 24A–D , 30A–C , 36 , 46 , 47

#### Material examined.

Total 8 specimens. ***Holotype***: 1♂, Tibet, Zayü, Zhowagoin, Xiongjiu village / 2022.7.13 / 28.60668°N, 97.28165°E, 1901 m / Hongbin Liang, Yuan Xu and Neng Zhang coll. (IZCAS); ***Paratypes***: 4♀1♂, Tibet, Zayü, Zhowagoin, Xiongjiu village / 2022.7.13 / 28.60668°N, 97.28165°E, 1901 m / Hongbin Liang, Yuan Xu and Neng Zhang coll. (IZCAS); 2♀, Tibet, Zayü, Zhowagoin, Xiongjiu village / 2021.7.1 / 28.60668°N, 97.28165°E, 1901 m / Hongbin Liang, Yuan Xu and Neng Zhang coll. (IZCAS).

#### Diagnosis.

Antennae nearly half as long as body length, antennomeres V–IX quadrate. Pronotum without distinct anterior and posterior transverse impression, pronotal disc with four or five fine punctures in middle. Elytral punctures sparse and absent on apical 1/3. Metasternal disc almost glabrous, posterior margin with sparse pubescence.

#### Description.

BL = 8.5–10.0 mm, BW = 3.8–4.5 mm. Head, most of legs, scutellum black, pronotum, elytra, femora of legs and underside brownish red.

**Figures 5–8.**
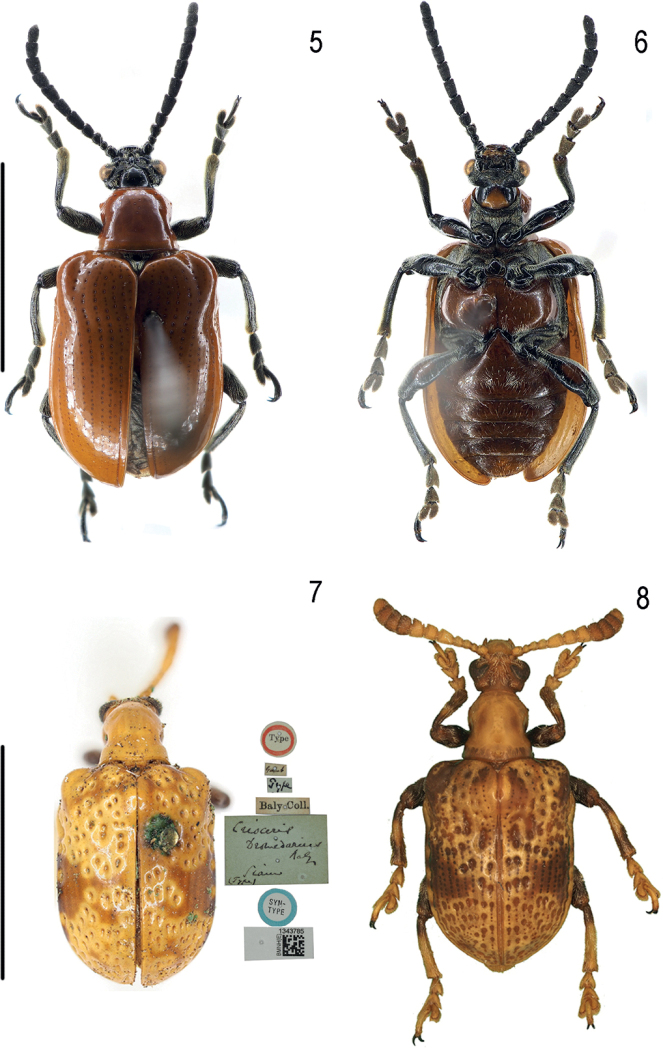
Habitus of *Lilioceris* spp. **5, 6***L.zayuensis*, holotype, Tibet **7***L.dromedarius*, syntype, Thailand **8***L.dromedarius*, specimen from Hainan. Scale bars: 5.0 mm.

***Head*** (Fig. 5). Vertex flat and without groove in middle, lateral side with sparse punctures and pubescence; clypeofrontal area triangular, lateral side of disc with sparse punctures and pubescence; labrum transverse, with sparse long pubescence; antennae nearly half as long as body, antennomeres I–IV nearly globular, antennomeres V–X 1.5 times as long as wide, III–XI densely pubescent and punctate.

***Pronotum*** (Figs 6, 21A). Anterior angle protruding, posterior angle not protruding; sides distinctly constricted in middle; without anterior and posterior transverse impression of disc; disc almost smooth and with four or five fine punctures in middle; basal transverse groove indistinct. Scutellum triangular and densely pubescent.

***Elytra*** (Fig. 5). Humeri protruding, humeral groove shallow, basal impression indistinct; striae with large punctures at base, punctures diminished posteriorly and absent on apical 1/3, intervals without punctures; epipleura raised, with row of fine punctures.

***Mesosternum*** pubescent, apical portion of mesosternal process strongly widened and convex, horizontally connected with metasternum (Fig. 21B); Metasternal disc almost glabrous, posterior margin with sparse pubescence. Metepisternum densely pubescent.

***Abdominal sternites*.** Lateral transverse impressions distinct on sternites I–IV, area of transverse impressions smooth, middle of sternite I with dense pubescence, other areas of sternites I–IV with sparse pubescence and punctures (Fig. 21C).

***Legs*** (Fig. 6). Femora with dense pubescence on dorsal surface, sparse pubescence on ventral surface, middle area widened.

***Male genitalia*** (Fig. 24A–D). Apical foramen occupying 1/5 length of median lobe (Fig. 24A); apex rounded (Fig. 24B); tegmen Y-shaped, basal piece of tegmen triangle and broad, lateral lobes slightly sclerotized and combined with second connecting membrane; internal sac with distinct dorsal, median, and ventral sclerites, posterior part of dorsal sclerite in dorsal view widened and rounded, ventral sclerite short and flat, median sclerite small (Fig. 24C, D).

***Female reproductive organs*** (Fig. 30A–C). Tergites VIII and IX, sternites VIII and IX sclerotized, posterior areas of tergite VIII and sternite VIII with pubescence and apodemes, spiculum gastrale Y-shaped and long; ovipositor with dense pubescence, distal part of ovipositor cylindrical, long and with protuberance; spermatheca greatly convoluted.

#### Distribution

**(Fig. 36).** China (Tibet).

#### Etymology.

The specific name *zayuensis* refers to its type locality Zayü County, Tibet, China.

#### Host plant and habitat

**(Figs 46, 47).** Beetles were collected feeding on *Smilaxlongebracteolata* J. D. Hooker (Smilacaceae) in Zayü. This species is confined to subtropical areas, at an elevation of ~ 1900 m. Xiongjiu village, Zayü has a mild climate and abundant rainfall. The habitat is secondary forest along a roadside, composed of tall trees, woody vines and many weeds.

#### Remarks.

This new species is a member of the *neptis* species group, and can be keyed out in couplet 4 with *L.cantonensis* (Heinze, 1943) and *L.neptis* (Weise, 1922) in the key by Xu et al. (2021: 301). It is different from *L.cantonensis* by its middle femora being completely black; elytral strial punctures dense and large at the base, diminished posteriorly, and absent on the apical 1/3 or 1/4; apical portion of the dorsal sclerite of the male genitalia is rounded. In *L.cantonensis*, the middle femora are bicolored, black with the middle brownish red on their ventral surfaces, and the elytral strial punctures sparse and large in basal impression, but absent on the apical 1/2 or 1/3; apical portion of the dorsal sclerite of the male genitalia truncated. It is also different from *L.neptis* by the black middle femora; metasternum almost glabrous, with only the posterior margin with sparse pubescence. In *L.neptis*, the middle femora are brownish red, and the metasternum has a long strip of setae extending from the anterior to the posterior margin.

This species is only found at the type locality. It seems that the local population was very low. We explored this place three times, but only collected eight specimens. We also tried several other places in Zayü County, but no specimens of this species were found.

##### ﻿New record for China

### ﻿ Lilioceris dromedarius

Taxon classificationAnimaliaColeopteraChrysomelidae

(Baly, 1861)

980829EB-2627-541F-BF9F-32B528E51704

Figs 7 , 8 , 31A–C , 37

Crioceris dromedarius Baly, 1861: 279 (Cambodia, syntype).
Lilioceris dromedarius : Monrós 1960: 175.
Crioceris rouyeri Pic, 1916: 18 (Java). Synonymized by Monrós 1960: 175.
Crioceris foveolata Pic, 1921: 33 (Cochinchina). Synonymized by Kimoto and Gressitt 1979: 221.

#### Type material examined.

1 ***syntype*** of *Liliocerisdromedarius* (NHML, photo), Type / Baly Coll. / *Criocerisdromedarius* Baly, Siam (Type) / SYNTYPE / BMNH(E)1345164.

#### Other material examined.

1 specimen. **Hainan**: 1♀, Wuzhi Shan, Shuiman township, Hudiegu (butterfly valley), 18.87482°N, 109.66819°E / 664 m, 2009.11.27, Meiying Lin coll. (IZCAS).

#### Diagnosis.

Antennae nearly half as long as body length, antennomeres VIII–X widened, twice as wide as long. Pronotal disc with two rows of fine punctures in middle. Elytra raised near suture at base, elytral punctures sparse and coarse, but absent at apex.

#### Redescription.

BL = 8.8 mm, BW = 4.5 mm. Antennomeres VIII–IX, head, femora and tibiae brownish, claws black, antennomeres I–VII, pronotum, elytra and abdomen brownish yellow, each elytron with big brownish marking in middle of lateral area, abdominal sternites except pygidium with three black markings in lateral side and middle.

***Head*** (Figs 7, 8). Vertex with groove in middle, punctate and pubescent densely; frontoclypeal area triangular, lateral side of disc with sparse punctures and pubescence; labrum transverse, with sparse long pubescence; antennae nearly 1/2 as long as body, antennomeres I–IV nearly globular, antennomere II shortest, antennomere V longest, antennomeres VI and VII length as long as width, antennomeres VIII–X widest, wide 2 times as long as length, antennomeres V–XI densely pubescent and punctate.

***Pronotum*** (Figs 7, 8). Anterior angle protruding, posterior angle not protruding; sides slight constricted in middle; middle of disc with two rows of fine punctures; anterior and posterior transverse impression indistinct, basal transverse groove very weak. Scutellum triangular and densely pubescent.

***Elytra*** (Figs 7, 8). Suture at base with raised hump; humeri protruding, humeral groove and basal impression distinct; striae with very sparse and coarse punctures, puncture absent at elytral brownish black marking area, intervals smooth; epipleura slightly raised, with row of fine punctures.

***Mesosternum*** pubescent; apical portion of mesosternal process narrow and flat, obliquely pointed, not horizontally connected with metasternum. Metasternal disc and metepisternum densely pubescent.

***Abdominal sternites*.** Lateral transverse impressions distinct on sternites I–IV, area of transverse impressions smooth, other areas with dense pubescence and punctures.

***Legs*.** Femora with dense pubescence on dorsal surface, with sparse pubescence on ventral surface, middle area widened.

***Male genitalia*.** Unknown.

***Female reproductive organs*** (Fig. 31A–C). Tergites VIII and IX, sternites VIII and IX sclerotized, posterior areas of tergite VIII and sternite VIII with pubescence and apodemes, spiculum gastrale Y-shaped and distinctly widen in distal part; ovipositor with dense pubescence, distal part of ovipositor cylindrical, long and with protuberance; spermatheca greatly convoluted.

#### Host plant and habitat.

A host plant is unknown. A single specimen in IZCAS was collected by Meiying Lin when beating vegetation. The collecting site, Hudiegu, is located in a tropical area in Wuzhi Shan of Hainan Province, with high temperatures, high humidity, and plentiful precipitation. Vegetation type is tropical evergreen broadleaved forest.

#### Distribution

**(Fig. 37).** China (Hainan); Vietnam; Thailand; Cambodia; Indonesia.

#### Remarks.

This species is very similar to *Liliocerisgibba* (Baly, 1861) (based on a syntype studied, NHML) but differs from the latter by antennomeres V–X being twice as wide as long (in *L.gibba*, antennomeres V–X as wide as long). In addition, the pronotum and elytra are yellow in *L.dromedarius* (dark brown in *L.gibba*).

##### ﻿New records

### ﻿ Lilioceris pulchella

Taxon classificationAnimaliaColeopteraChrysomelidae

(Baly, 1859)

79A2BD63-F894-58F1-AE98-87892A8133C3

**Figures 9–12.**
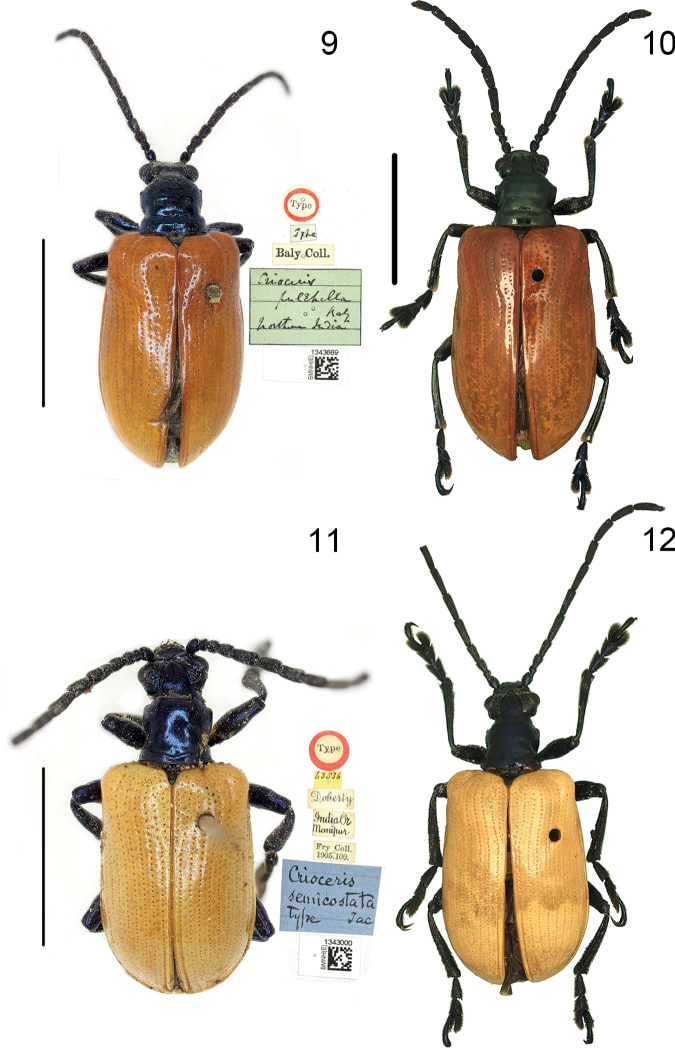
Habitus of *Lilioceris* spp. photographed by HBL **9, 10***L.pulchella*, type, India **11, 12***L.semicostata*, type, India (Manipur). Scale bars: 5.0 mm.

Figs 9 , 10 , 32A–C , 37

Crioceris pulchella Baly, 1859: 152 (India, syntype).
Lilioceris pulchella : Monrós 1960: 171.

#### Type material examined.

1 ***syntype*** of *Liliocerispulchella* (NHML, photo), Type / Baly Coll. / *Criocerispulchella* Baly, India (Type) / BMNH(E) 1343669.

#### Other material examined.

1 specimen. **Tibet**: 1♀, Hanmi–Lage, 2005.08.28, Dakang Zhou coll. (IZCAS).

#### Diagnosis.

Antennae ~ 1/3 as long as body, antennomeres V–X cylindrical. Pronotum with distinct posterior transverse impression, pronotal disc with two rows of fine punctures in middle, scutellum pubescent. Elytral punctures fine, diminishing posteriorly, but not absent. Lateral side of metasternite with long narrow strip of pubescence.

#### Redescription.

BL = 11.0 mm, BW = 5.0 mm. Antennae, head, pronotum, scutellum, prosternum, mesosternum, legs, and half of first abdominal sternite black, with blue metallic luster, elytra and remainder of abdominal sternite brownish red.

***Head*** (Figs 9, 10). Vertex with deep groove in middle, sparsely punctate and pubescent in lateral area; frontoclypeal area triangular, lateral side of disc with sparse punctures and pubescence; labrum transverse, middle of anterior margin concave, disc with sparse punctures and pubescence; antennae nearly 3/5 length of body, antennomeres I–IV nearly globular, antennomere II shortest, antennomeres V–XI cylindrical, 3 times as long as wide.

***Pronotum*** (Figs 9, 10). Anterior angle protruding, posterior angle not protruding; sides constricted in middle; anterior and posterior transverse impression distinct; disc with irregular fine punctures in middle; scutellum triangular and densely pubescent.

***Elytra*** (Figs 9, 10). Humeri protruding, humeral groove distinct, basal impression indistinct; striae with fine punctures, diminishing posteriorly and absent at end, intervals smooth; epipleura raised, with row of fine punctures.

***Mesosternum*** pubescent; apical portion of mesosternal process narrow and flat, obliquely pointed, not horizontally connected with metasternum. Lateral side of metasternite with a long narrow strip of pubescence extending from the lateroposterior corner to anterior margin. Metepisternum densely pubescent.

***Abdominal sternites*.** Lateral transverse impressions indistinct on sternites I–IV. Lateral side of sternite I–IV with densely pubescent, other areas with sparse pubescence and punctures.

***Legs*.** Femora with dense pubescence in dorsal surface, with sparse pubescence on ventral surface, middle area widened. Claws distinctly asymmetrical, outer one is longer than inner one.

***Male genitalia*.** Unknown.

***Female reproductive organs*** (Fig. 32A–C). Tergites VIII and IX, sternites VIII and IX sclerotized, posterior areas of tergite VIII and sternite VIII with pubescence and apodemes, spiculum gastrale X-shaped and short; ovipositor with dense pubescence, distal part of ovipositor cylindrical, short, and with protuberance; spermatheca simple and folded.

#### Distribution

**(Fig. 37).** China (Tibet); India.

#### Host plant and habitat.

A host plant is unknown. This species was collected in an environment between Hanmi and Lage according to the collector. Hanmi and Lage are two small courier stations on an old trail from Doxong La pass to Baibung town in Mêdog County, at altitudes of 2000–3000 m. They are located in a subtropical area, with a primary forest composed of large trees, woody vines and many shrubs.

#### Remarks.

A single specimen found in IZCAS was collected by Mr Dakang Zhou with a sweeping net in 2005. In recent years, we have surveyed Hanmi, Mêdog several times, but no more specimens have been found.

### ﻿ Lilioceris semicostata

Taxon classificationAnimaliaColeopteraChrysomelidae

(Jacoby, 1908)

89617BD4-0B68-5249-9E16-DCA17BF5370F

Figs 11 , 12 , 25A–D , 33A–C , 37

Crioceris semicostata Jacoby, 1908: 77 (India: Manipur, syntype).
Lilioceris semicostata : Monrós 1960: 179.

#### Type material examined.

1 ***syntype*** of *Liliocerissemicostata* (NHML, photo), Type /63836 /Doherty / India Or. Manipuria / Frey Coll., 1905.100. / *Criocerissemicostata*, Type, Jac / BMNH(E)1343000.

#### Other material examined.

2 specimens. **Tibet**: 1♀, Mêdog, Baibung Town, Hanmi / 29.36739°N, 95.12728°E, 2123 m, 2011.07.24 / Ye Liu coll. (IZCAS); 1♂, China, Tibet, Mêdog, Baibung, Hanmi / host unknown / 29.3664°N, 95.1277°E, 2120 m, 2011.07.26 / Xiaodong Yang coll. (IZCAS).

#### Diagnosis.

Antennae nearly as long as body, antennomeres V–X cylindrical. Pronotum with distinct posterior transverse impression, pronotal disc almost smooth, scutellum pubescent. Elytral punctures diminishing posteriorly, but not absent. Metasternite with scattered and sparse pubescence.

#### Redescription.

BL = 12.0–15.0 mm, BW = 4.0–6.0 mm. Elytra yellow, rest of body black and with blue metallic luster.

***Head*** (Figs 11, 12). Vertex raised, with deep groove in middle, sparsely punctate and pubescent in lateral area; frontoclypeal area triangular, lateral side of disc with sparse punctures and pubescence; labrum transverse, middle of anterior margin concave, disc with sparse punctures and long pubescence; antennae nearly as long as body length, antennomeres I–III nearly globular, antennomere II shortest, antennomeres IV–XI cylindrical, 3 times as long as wide.

***Pronotum*** (Figs 11, 12). Anterior angle protruding, posterior angle not protruding; sides slightly constricted in middle; posterior transverse impression distinct, disc almost smooth; scutellum triangular and smooth.

***Elytra*** (Figs 11, 12). Humeri protruding, humeral groove distinct, basal impression indistinct; striae with fine punctures, punctures diminishing posteriorly but not absent, intervals smooth; epipleura raised, with row of fine punctures.

***Mesosternum*** pubescent; apical portion of mesosternal process narrow and flat, obliquely pointed, not horizontally connected with metasternum. Metasternite with scattered and sparse pubescence. Metepisternum sparsely pubescent.

***Abdominal sternites*.** Lateral transverse impressions indistinct on sternites I–IV. Lateral sides of sternites I–IV with denser pubescence, other areas with sparse or scattered pubescence.

***Legs*.** Femora with dense pubescence on dorsal surface, with sparse pubescence on ventral surface, middle area widened. Claws distinctly asymmetrical, outer one longer than inner one.

***Male genitalia*** (Fig. 25A–D). Apical foramen occupying 1/5 length of median lobe (Fig. 25A); apex truncated (Fig. 25B); tegmen Y-shaped, basal piece of tegmen triangular and broad, lateral lobes slightly sclerotized and combined with second connecting membrane; internal sac with distinct dorsal, median and ventral sclerites, posterior part of dorsal sclerite in dorsal view widen, ventral sclerite short and flat, median sclerite distinct (Fig. 25C, D).

***Female reproductive organs*** (Fig. 33A–C). Tergites VIII and IX, sternites VIII and IX sclerotized, posterior areas of tergite VIII and sternite VIII with pubescence and apodemes, spiculum gastrale X-shaped and long; ovipositor with dense pubescence, distal part of ovipositor cylindrical, short and with protuberance; spermatheca simple and curved.

#### Distribution

**(Fig. 37).** China (Tibet); India.

#### Host plant and habitat.

Host plant is unknown. Specimens of this species in IZCAS were collected in Hanmi by Ye Liu and Xiaodong Yang when sweeping the vegetation canopy. Hami is located in the subtropical area of Mêdog, with primary forest composed of large trees, woody vines and many shrubs.

#### Remarks.

This species is very similar to *Liliocerisflavipennis* (Baly, 1859) (based on a syntype studied, NHML), but differs from the latter by the pronotum being slightly constricted in middle, anterior and posterior angles not protruding; elytral striae regular. In *L.flavipennis*, pronotum is strongly constricted in middle, anterior and posterior angles are strongly protruding; elytral striae are irregular.

### ﻿ Lilioceris unicolor

Taxon classificationAnimaliaColeopteraChrysomelidae

(Hope, 1831)

8D9F1921-764F-534E-9AD7-3CB57BF8E7C3

**Figures 13–18.**
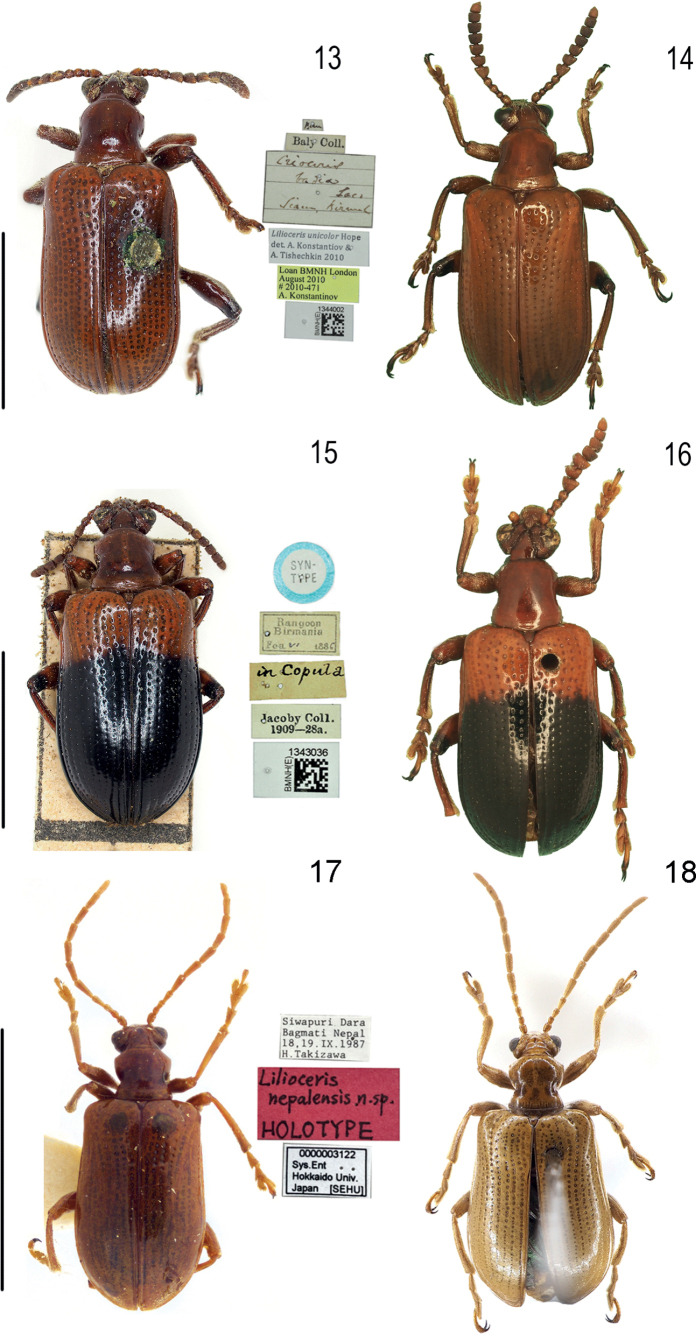
Habitus of *Lilioceris* spp. **13***L.unicolor*, specimen from Thailand **14***L.unicolor*, specimen from China (Yunnan) **15***L.seminigra*, syntype, Myanmar **16***L.seminigra*, specimen from Yunnan **17***L.nepalensis*, holotype, Nepal (Bagmati), photographed by Takuya Takemoto **18***L.nepalensis*, specimen from China (Dinggyê) Scale bars: 5.0 mm.

Figs 13–16 , 26A–D , 35A–C , 37

Crioceris unicolor Hope, 1831: 28 (Nepal, lectotype).
Lilioceris unicolor : Monrós 1960: 172.
Crioceris badia Lacordaire, 1845: 560 (Siam. Type not found). Synonymized by Clavareau 1913: 51.
Crioceris seminigra Jacoby, 1889: 153 (Birma: Tenasserim, syntype). syn. nov.
Lilioceris seminigra : Monrós 1960: 171.

#### Type material examined.

1♂ ***lectotype*** of *Liliocerisunicolor* (NHML, photo, antennae, sternites middle and hind legs missing), Type / Nepal / Hardwicke, Bequest / unicolor Hope / Lectotype, *Liliocerisunicolor* (Hope) des. A. Konstantinov & A. Tishechkin 2010 / Loan BMNH August 2010 # 2010-471/ BMNH(E)1343990; 1 ***syntype*** of *Liliocerisseminigra* (NHML, photo), Rangoon, Brimania, Fea. VI. 1886/ in copula / Jacoby Coll., 1909–28a. / BMNH(E), 1343036.

#### Other material examined.

5 specimens. **Thailand**: 1 specimen of *Liliocerisunicolor* (NHML, photo): Siam/ Baly coll./ *Criocerisbadia* Lac. Siam “illegible”/ *Liliocerisunicolor* Hope, det. A. Konstantinov and Tishechkin 2010/ Loan BMNH August 2010 # 2010-471 A. Konstantinov/ BMNH (E)1344002; 1♀, locality is illegible / *Criocerisseminigra* Jac. var. (handwriting seems Jacoby’s), (IZCAS); **Yunnan**: 1♀, Longchuan / 1150 m, 1979.VII.16 / *Liliocerisseminigra* Jacoby, det. Peiyu Yu, (IZCAS); 1♀, Yunnan, Yingjiang, Nongzhang, Jiemao, Xianrendong, 24.52567°N, 97.79818°E / 837 m, 2020.5.27, Hongbin Liang and Yuan Xu coll. (IZCAS); 1♂, Longchuan, Jinghan, Longbazhai, S223 road/ 24.27601°N, 97.85190°E, 902 m, 2020.5.26, Hongbin Liang and Yuan Xu coll. (IZCAS).

#### Diagnosis.

Antennae ~ 1/3 as long as body, antennomeres V–XI quadrate. Pronotum without anterior and posterior transverse impression, pronotal disc with one or two rows of punctures in middle. Elytral punctures large, not diminishing posteriorly, intervals slightly convex on apical 1/4; epipleura raised, with row of fine punctures.

#### Redescription.

BL = 8.0 mm, BW = 3.8 mm. Body brownish red.

***Head*** (Figs 13–16). Vertex with shallow groove in middle, sparsely punctate and pubescent in lateral area; frontoclypeal area triangular, disc with sparse punctures and pubescence; labrum transverse, disc with sparse long pubescence; antennae nearly 1/3 length of body, antennomeres I–IV nearly globular, antennomere II shortest, antennomeres V–X strongly widened, 2 times as wide as long.

***Pronotum*** (Figs 13–16). Anterior angle protruding, posterior angle not protruding; sides constricted in middle; anterior and posterior transverse impression absent; middle of disc with one or two rows of fine punctures. Scutellum triangular and pubescent.

**Figures 19–21.**
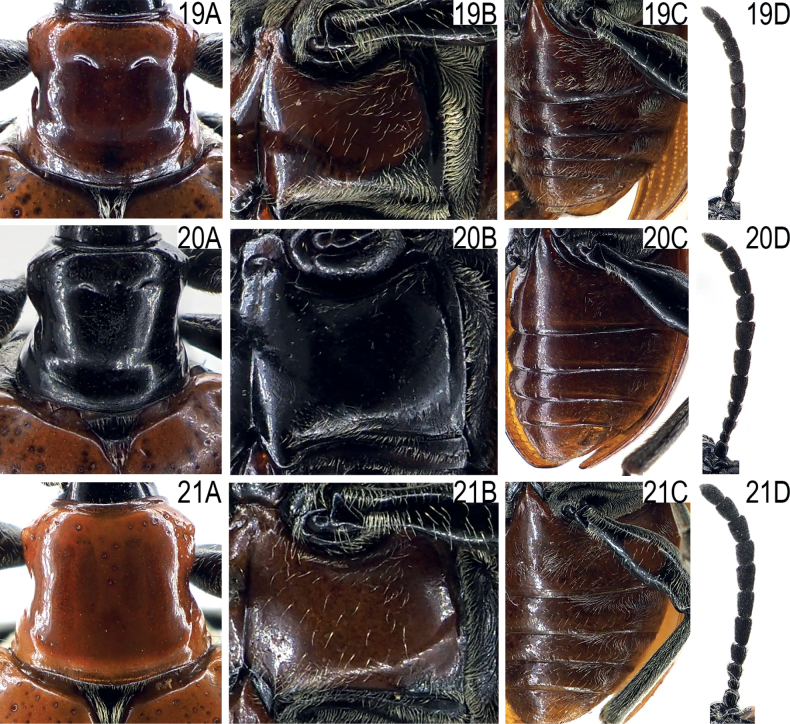
Pronotum, mesosternal disc, abdominal sternites and antennae of *Lilioceris* spp. **19***L.zhentangensis*, ♂, China (Tibet: Zhêntang) **20***L.medogensis*, ♂, China (Tibet: Mêdog) **21***L.zayuensis*, ♂, China (Tibet: Zayü) **A** pronotum **B** mesosternal disc **C** abdominal sternite **D** antennae.

***Elytra*** (Figs 13–16). Humeri protruding, humeral groove distinct, basal impression indistinct; striae with large punctures, punctures not diminishing posteriorly, intervals slightly convex on apical 1/4; epipleura raised, with row of fine punctures.

**Figures 22–24.**
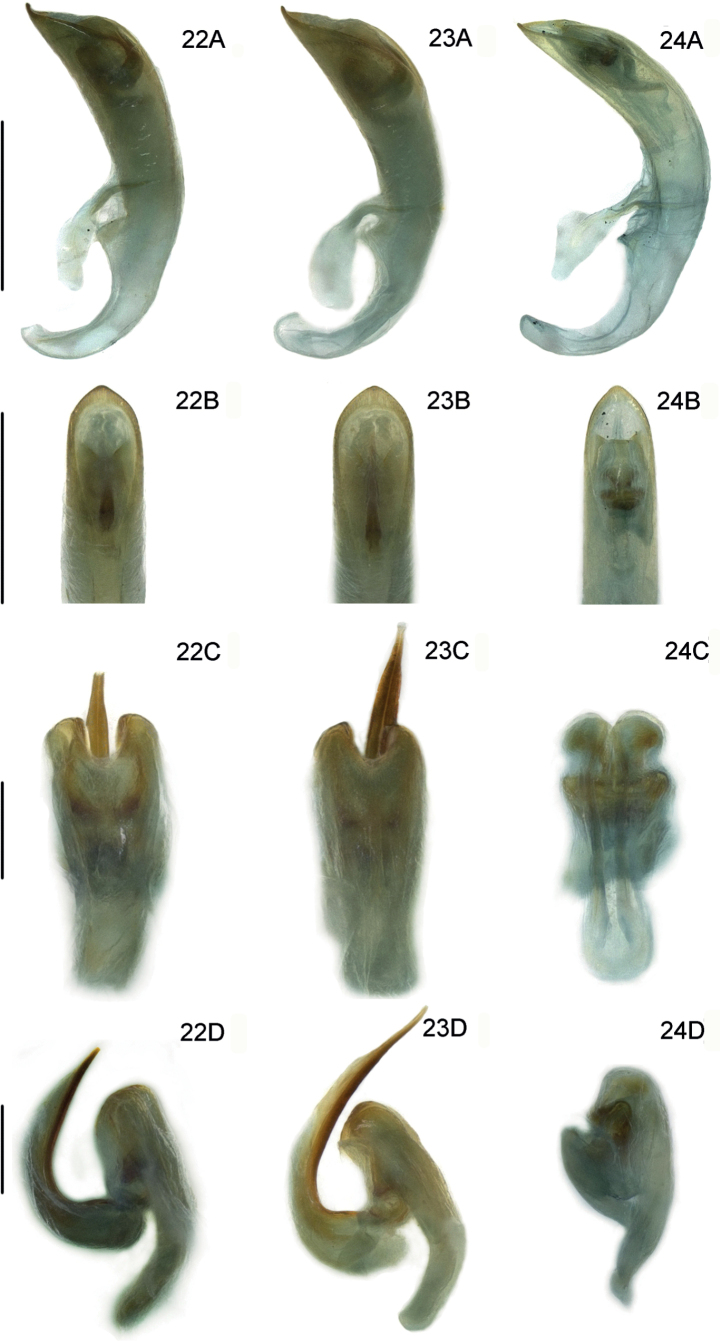
Male genitalia of three new *Lilioceris* species (holotypes) **22***L.zhentangensis*, China (Tibet: Dinggyê) **23***L.medogensis*, China (Tibet: Mêdog) **24***L.zayuensis*, China (Tibet: Zayü) **A** aedeagus, lateral view **B** aedeagus, dorsal view **C** dorsal sclerite, dorsal view **D** sclerites in internal sac, lateral view. Scale bars: 0.5 mm (**A, B**); 0.2 mm (**C, D**).

***Mesosternum*** pubescent; apical portion of mesosternal process widened and flat, obliquely pointed, not horizontally connected with metasternum. Metasternum with long strip of pubescence along outer side, extending from anterior to posterior margin. Metepisternum densely pubescent.

***Abdominal sternites*.** Lateral transverse impressions indistinct on sternites I–V, lateral sides of sternites I–V with dense pubescence, only with sparse pubescence in middle.

***Legs*.** Femora with dense pubescence on dorsal surface, with sparse pubescence on ventral surface, middle area widened.

***Male genitalia*** (Fig. 26A–D). Apical foramen occupying 1/5 length of median lobe (Fig. 26A); apex hooked (Fig. 26B); tegmen Y-shaped, basal piece of tegmen triangle and broad, lateral lobes slightly sclerotized and combined with second connecting membrane; internal sac with distinct dorsal, median and ventral sclerites, posterior part of dorsal sclerite in dorsal view widen and anterior part of dorsal sclerite distinctly extended, ventral sclerite short and flat, median sclerite distinct (Fig. 26C, D).

**Figures 25–27.**
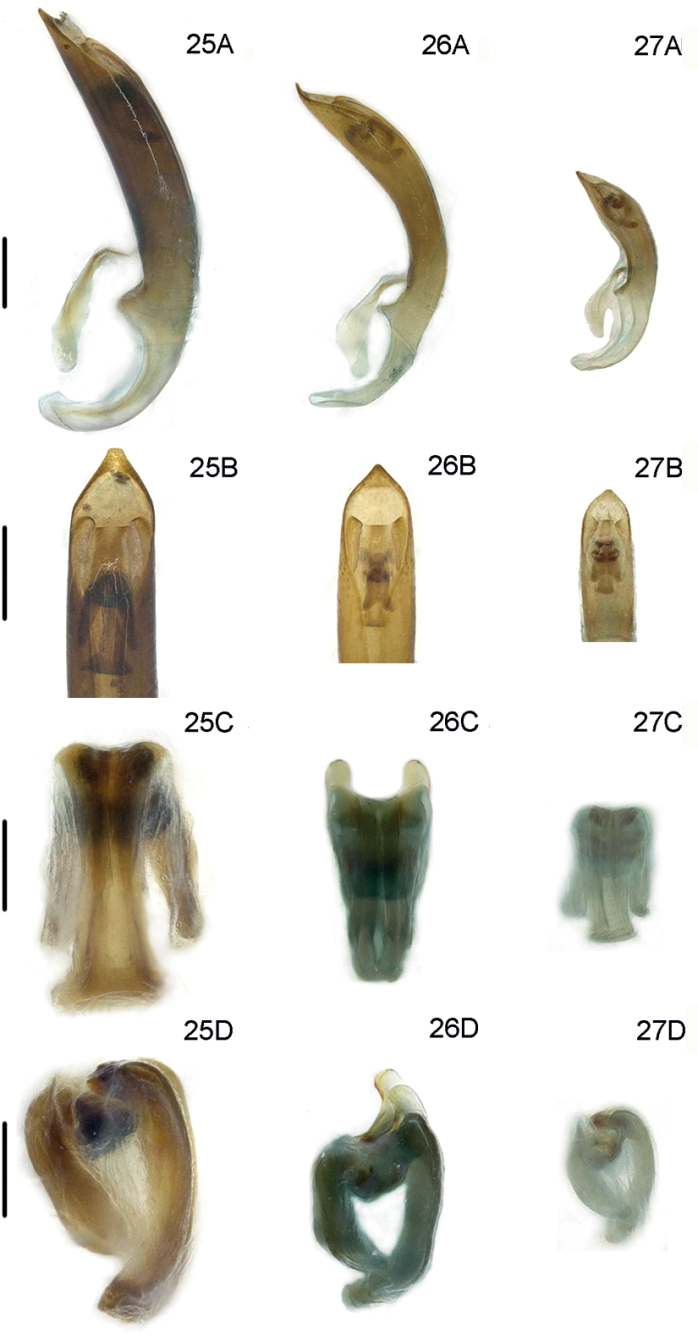
Male genitalia of new records of *Lilioceris* species in China **25***L.semicostata*, China (Tibet: Mêdog) **26***L.unicolor*, China (Yunnan: Longchuan) **27***L.nepalensis*, China (Tibet: Dinggyê) **A** aedeagus, lateral view **B** aedeagus, dorsal view **C** dorsal sclerite, dorsal view **D** sclerites in internal sac, lateral view. Scale bars: 0.5 mm (**A, B**); 0.2 mm (**C, D**).

***Female reproductive organs*** (Fig. 35A–C). Tergites VIII and IX, sternites VIII and IX sclerotized, posterior areas of tergite VIII and sternite VIII with pubescence and apodemes, spiculum gastrale Y-shaped and long; ovipositor with dense pubescence, distal part of ovipositor cylindrical, short and with protuberance; spermatheca convoluted.

#### Distribution

**(Fig. 37).** China (Yunnan); Nepal, Myanmar.

#### Host plant and habitat.

We collected two adults of *L.unicolor* on *Dioscorea* sp. (Dioscoreaceae) in the villages of Jiemao and Longbazhai of Yunnan Province, but feeding was not observed, so their host plant needs confirmation. Jiemao and Longbazhai are located in a subtropical area, at elevations of 800–900 m. These two places have a mild climate and abundant rainfall. The habitat in Jiemao is a large secondary forest, composed of tall trees, woody vines, shrubs and weeds. The habitat in Longbazhai is a very small secondary forest of several thousand square meters, surrounded by crop fields.

#### Remarks.

We examined the lectotype and a non-type specimen of *L.unicolor* present at NHML. The specimens in IZCAS from Yunnan are not significantly different from the lectotype. The punctures on pronotum are variable: the lectotype has two rows of punctures in the middle of the pronotum, the non-type specimen in NHML and the specimen in IZCAS have only one row of punctures in the middle of the pronotum. The male genitalia of our specimen from Yunnan (Fig. 26C, D) are identical to those of the lectotype in NHML (Tishechkin et al. 2011: 80, fig. 30).

We also examined a syntype of *L.seminigra* in NHML, and no significant morphological difference was found from the lectotype of *L.unicolor*, except for the bi-coloration on the elytra of *L.seminigra*. When Jacoby (1889: 153) described *L.seminigra*, he noticed a variant: “Var. Elytra entirely fulvous”. In four Chinese specimens in IZCAS, the elytrae are all brownish-red in two specimens of *L.unicolor*; but the apical 2/3 of each elytron is black, 1/3 of base is brownish-red in one specimen of *L.seminigra*, and completely brownish-red in another specimen. The female reproductive organs of these two species were dissected and compared, and no significant differences were found. Therefore, we conclude that *L.unicolor* and *L.seminigra* are conspecific, and elytral bi-coloration in *L.seminigra* is simply a variation. In addition, antennomeres V–X in this species are flat, strongly transverse, and the outer distal angle of antennomeres V–VII is protruding. These characteristics are unique among *Lilioceris* species.

### ﻿ Lilioceris nepalensis

Taxon classificationAnimaliaColeopteraChrysomelidae

Takizawa, 1989

FB4CCE24-D01A-5C9A-B65E-AEB2AC114B4F

Figs 17 , 18 , 27A–D , 34A–C , 37 , 48–51

Lilioceris nepalensis Takizawa, 1989: 327 (Nepal: Bagmati, holotype).

#### Type material examined.

***Holotype*** of *Liliocerisnepalensis* (SEHU, photo), *Liliocerisnepalensis* n.sp., Holotype / Siwapuri Dara Bagmati Nepal 18, 19.IX.1987, H. Takizawa / 0000003122, Sys. Ent Hokkaido Univ. Japan [SEHU].

#### Other material examined.

32 specimens. **Tibet**: 9♀12♂, Dinggyê, Zhêntang, Nadang village, 27.85317°N, 87.44903°E / 2491 m, 2021.6.25, Hongbin Liang, Yuan Xu and Neng Zhang coll. (IZCAS); 4♀2♂, Jilong, near to Zhaoti Bilei, 28.49216°N, 85.22383°E / 3219 m, 2023.7.16, Yuyao Qin and Yong Wang coll. (IZCAS); 2♀3♂, Cona, 1 km north of Mama township, 27.89875°N, 91.80188°E / 2939 m, 2023.9.2–3, Hongbin Liang coll. (IZCAS).

#### Diagnosis.

Antennae ~ 2/3 as long as body, antennomeres V–X cylindrical. Pronotum with distinct posterior transverse impression, pronotal disc smooth. Elytral punctures sparse on basal half but absent on apical half. Metasternite smooth.

#### Redescription.

BL = 4.8.0–6.5 mm, BW = 3.0–4.0 mm. Body almost brownish red, only sternum and abdominal sternites I and II black.

***Head*** (Figs 17, 18). Vertex flat, with shallow groove in middle, punctate and pubescent in lateral area; frontoclypeal area triangular, disc with fine punctures and sparse pubescence; labrum transverse, with sparse pubescence; antennae 2/3 length of body, antennomeres I–III nearly globular, antennomeres IV–XI cylindrical, antennomeres V–XI 3 times as long as wide.

***Pronotum*** (Figs 17, 18). Anterior angle protruding; posterior angle not protruding; sides distinctly constricted in middle; posterior transverse impression distinct, disc almost smooth; scutellum triangular and smooth.

***Elytra*** (Figs 17, 18). Humeri protruding, humeral groove distinct, basal transverse impression indistinct; strial punctures large at base, diminishing posteriorly and absent on apical 1/2, intervals smooth; epipleura raised, with row of fine punctures.

***Mesosternum*** pubescent; apical portion of mesosternal process narrow and flat, obliquely pointed, not horizontally connected with metasternum. Metasternum and metepisternum smooth.

***Abdominal sternites.*** Lateral transverse impressions absent on sternites I–IV. Sternites I–IV smooth.

***Legs*.** Femora with dense pubescence on dorsal surface, with sparse pubescence on ventral surface. Claws distinctly asymmetrical, outer one longer than inner one.

***Male genitalia*** (Fig. 27A–D). Apical foramen occupying 1/5 length of median lobe (Fig. 27A); apex rounded (Fig. 27B); tegmen Y-shaped, basal piece of tegmen triangle and broad, lateral lobes slightly sclerotized and combined with second connecting membrane; internal sac with distinct dorsal, median and ventral sclerites, posterior part of dorsal sclerite in dorsal view slightly widen, ventral sclerite short and flat, median sclerite distinct (Fig. 27C, D).

**Figures 28–30.**
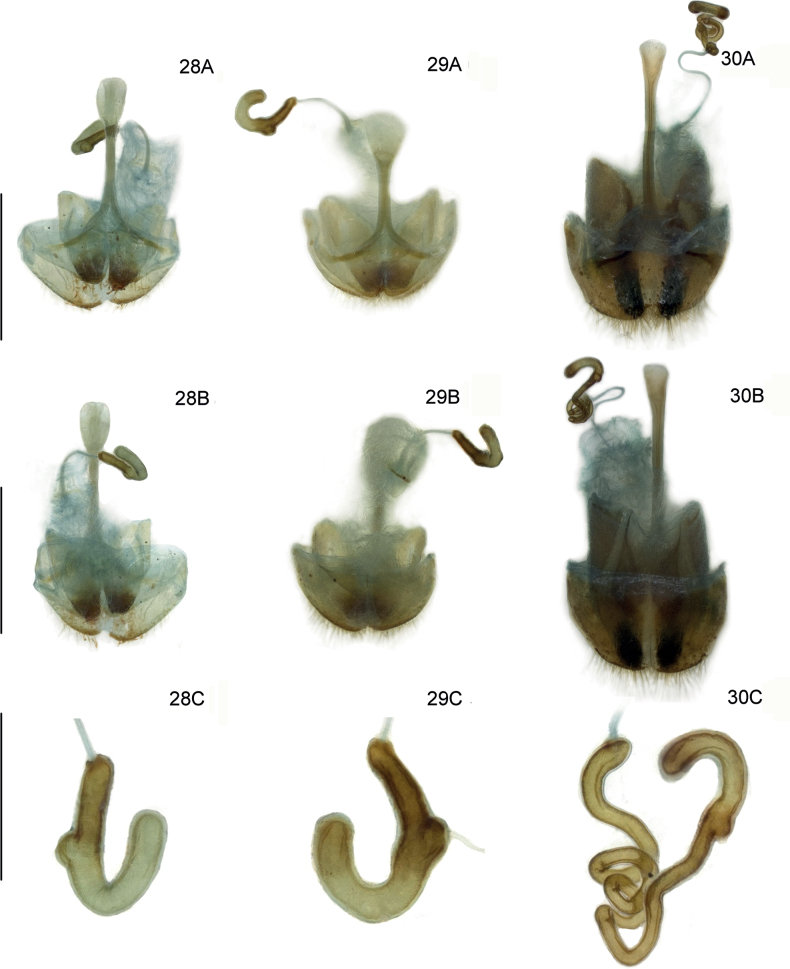
Female reproductive organs of three new *Lilioceris* species (paratypes) **28***L.zhentangensis*, China (Tibet: Dinggyê) **29***L.medogensis*, China (Tibet: Mêdog) **30***L.zayuensis*, China (Tibet: Zayü) **A** dorsal view **B** ventral view **C** spermatheca. Scale bars: 0.5 mm.

***Female reproductive organs*** (Fig. 34A–C). Tergites VIII and IV, sternites VIII and IV sclerotized, posterior areas of tergite VIII and sternite VIII with pubescence and apodemes, spiculum gastrale X-shaped and short; ovipositor with dense pubescence, distal part of ovipositor cylindrical, short and with protuberance; spermatheca greatly convoluted.

**Figures 31–35.**
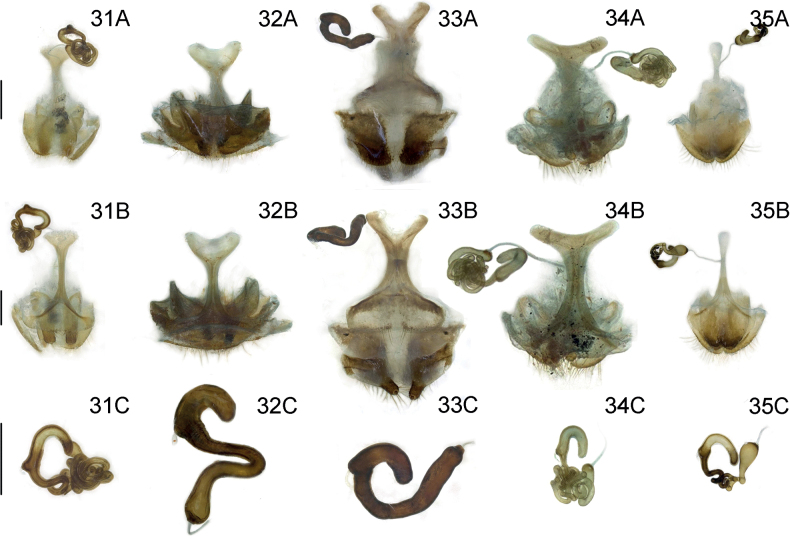
Female reproductive organs of five new records of *Lilioceris* species in China **31***L.dromedarius*, China (Hainan: Wuzhi Shan) **32***L.pulchella*, China (Tibet: Mêdog) **33***L.semicostata*, China (Tibet: Mêdog) **34***L.nepalensis*, China (Tibet: Dinggyê) **35***L.unicolor*, China (Yunnan: Yingjiang) **A** dorsal view **B** ventral view **C** spermatheca. Scale bars: 0.5 mm (**A–C**).

**Figure 36.**
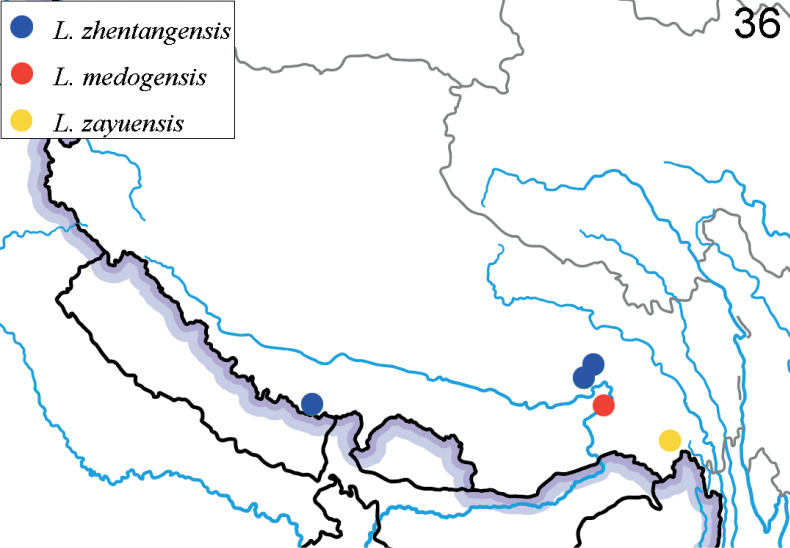
Collecting sites of three new *Lilioceris* species.

#### Distribution

**Figure 37.**
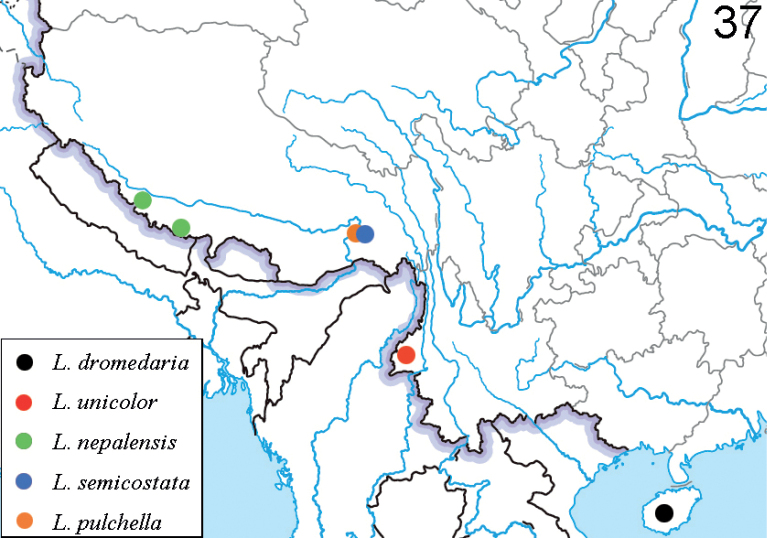
Collecting sites of five new records of *Lilioceris* species in China (distributions outside China are not marked here because of the lack of precise locality data).

**(Fig. 37).** China (Tibet); Nepal; India.

**Figures 38–41.**
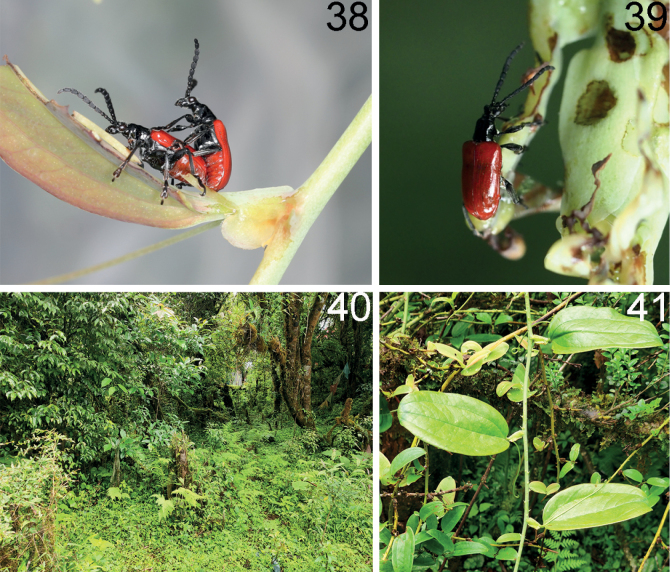
*Liliocerismedogensis* in Tibet (Mêdog), 2021.VI.9 **38, 39** adult **40** habitat **41** host plant: *Smilaxferox***38** photographed by HBL **39–41** photographed by YX.

**Figures 42–45.**
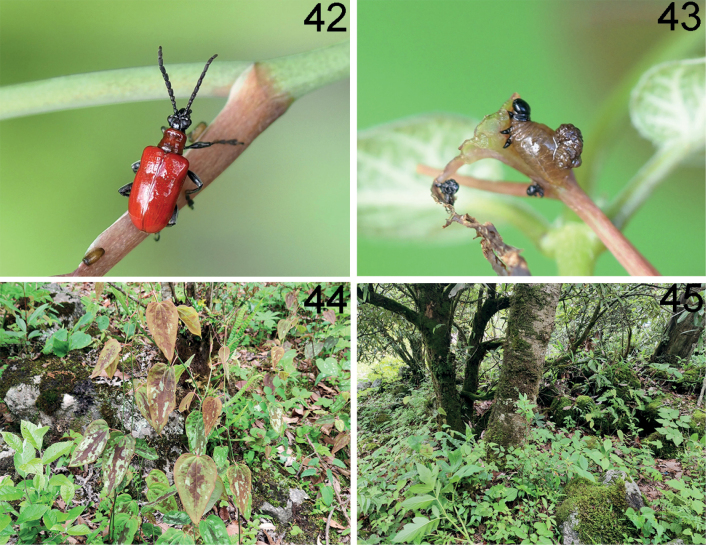
*Lilioceriszhentangensis* in Tibet (Dinggyê), 2021.VI.24, photographed by YX **42** adult **43** larva **44** host plant: *Smilaxmenispermoidea***45** habitat.

**Figures 46, 47.**
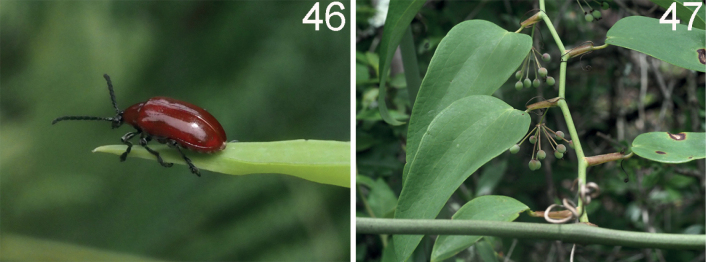
*Lilioceriszayuensis* in Tibet (Zayü), 2022.VII.13, photographed by YX **46** adult **47** host plant: *Smilaxlongebracteolata*.

#### Host plant and habitat

**Figures 48–51.**
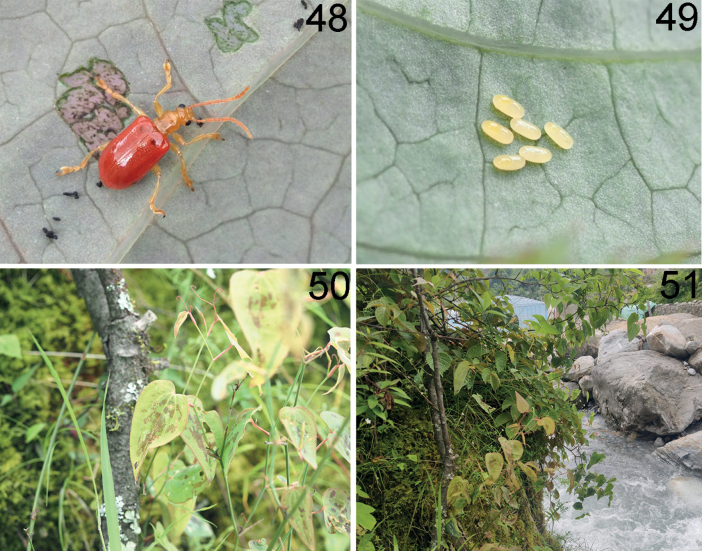
*Liliocerisnepalensis* in Tibet (Zhêntang), 2021.VI.24, photographed by YX **48** adult **49** egg **50** host plant: *Smilaxmenispermoidea***51** habitat.

**(Figs 48–51).** The host plant of this species is *Smilaxmenispermoidea* A. DC. (Smilacaceae) according to our observations in Dinggyê. *Liliocerisnepalensis* was found on its host plant near rivers at altitudes of 2400 to 3200 m, sharing the same habitat with *L.zhentangensis* in Nadang village of Zhêntang, Dinggyê.

#### Remarks.

This species is only ~ 5.0 mm long. It shares the following characteristics with *L.pulchella*, *L.semicostata*, *L.flavipennis* and *L.adonis* (Baly, 1859) (based on a syntype studied, NHML): antennae length > 1/2 of body length, antennomeres IV–X cylindrical, 3 times as long as wide; claws distinctly asymmetrical, outer one longer than inner one.

The black spots on the elytra are actually black spots on the exoskeleton covering the flight muscles under the elytra. These black muscles can be seen clearly when this insect is alive (Fig. 48), but when it is dead, they may be visible as spots (Fig. 17) or not (Fig. 18).

## Supplementary Material

XML Treatment for Lilioceris zhentangensis

XML Treatment for Lilioceris medogensis

XML Treatment for Lilioceris zayuensis

XML Treatment for Lilioceris dromedarius

XML Treatment for Lilioceris pulchella

XML Treatment for Lilioceris semicostata

XML Treatment for Lilioceris unicolor

XML Treatment for Lilioceris nepalensis

## ﻿Additional information

### Data availability

All of the data that support the findings of this study are available in the main text.
